# Protocol for implementation of family health history collection and decision support into primary care using a computerized family health history system

**DOI:** 10.1186/1472-6963-11-264

**Published:** 2011-10-11

**Authors:** Lori A Orlando, Elizabeth R Hauser, Carol Christianson, Karen P Powell, Adam H Buchanan, Blair Chesnut, Astrid B Agbaje, Vincent C Henrich, Geoffrey Ginsburg

**Affiliations:** 1 Department of Medicine, Duke University, 3475 Erwin Rd, Durham, NC, 27705, USA; 2 Center for Human Genetics, Duke University, Box 3445, Durham, NC, 27710, USA; 3 Center for Biotechnology, Genomics, and Health Research, University of North Carolina at Greensboro, 1111 Spring Garden St, Greensboro, NC, 27412, USA; 4 Duke Cancer Institute, Duke University, 2424 Erwin Road, Durham, NC, 27705, USA, Center for Human Genetics, Duke University, Box 3445, Durham, NC, 27710, USA; 5 Business Development Office, 1200 N. Elm Street, Moses Cone Health System, Greensboro, NC, 27401, USA; 6 Center for Personalized Medicine, Duke University, 101 Science Drive, Durham, NC, 27708, USA

## Abstract

**Background:**

The CDC's Family History Public Health Initiative encourages adoption and increase awareness of family health history. To meet these goals and develop a personalized medicine implementation science research agenda, the Genomedical Connection is using an implementation research (T3 research) framework to develop and integrate a self-administered computerized family history system with built-in decision support into 2 primary care clinics in North Carolina.

**Methods/Design:**

The family health history system collects a three generation family history on 48 conditions and provides decision support (pedigree and tabular family history, provider recommendation report and patient summary report) for 4 pilot conditions: breast cancer, ovarian cancer, colon cancer, and thrombosis. All adult English-speaking, non-adopted, patients scheduled for well-visits are invited to complete the family health system prior to their appointment. Decision support documents are entered into the medical record and available to provider's prior to the appointment. In order to optimize integration, components were piloted by stakeholders prior to and during implementation. Primary outcomes are change in appropriate testing for hereditary thrombophilia and screening for breast cancer, colon cancer, and ovarian cancer one year after study enrollment. Secondary outcomes include implementation measures related to the benefits and burdens of the family health system and its impact on clinic workflow, patients' risk perception, and intention to change health related behaviors. Outcomes are assessed through chart review, patient surveys at baseline and follow-up, and provider surveys. Clinical validity of the decision support is calculated by comparing its recommendations to those made by a genetic counselor reviewing the same pedigree; and clinical utility is demonstrated through reclassification rates and changes in appropriate screening (the primary outcome).

**Discussion:**

This study integrates a computerized family health history system within the context of a routine well-visit appointment to overcome many of the existing barriers to collection and use of family history information by primary care providers. Results of the implementation process, its acceptability to patients and providers, modifications necessary to optimize the system, and impact on clinical care can serve to guide future implementation projects for both family history and other tools of personalized medicine, such as health risk assessments.

## Background

In 2002 the CDC launched the Family History Public Health Initiative, founded upon the principle that family history is an under used but effective tool for risk stratification. Two of the stated goals are to develop tools to enhance family health history (FHH) collection and to evaluate whether family history-based strategies work. Given that primary care providers are the first (and sometimes only) provider most patients see and are long-term partners and coordinators in their care, primary care practices play a crucial role in collecting and integrating FHH into an individual's personalized health plan. They, therefore, are a natural choice as partners in studying the implementation of family history collection into the medical decision making process.

Although FHH is considered a standard component of the medical interview and several guidelines tie screening strategies to risk based upon family history, substantial barriers exist to widespread adoption in clinical practice. Barriers can be broadly categorized into those related to collecting family history and those related to acting upon family history. Among those related to collecting family history are: 1) most primary care practices do not have the resources or the time to collect a complete FHH or to update it [1]; 2) many patients are either unaware or have limited knowledge about their FHH and are therefore not able to answer questions accurately and completely at the point of care [2]; and 3) many providers do not have adequate training and thus fail to recognize all but the most straightforward patterns related to inherited syndromes [3,4]. Among the barriers related to acting upon family history are the lack of clear cut recommendations about how to manage moderate and high risk patients, and the lack of adequate communication and support from family history specialists, genetic counselors, and subspecialists that are necessary to provide appropriate management.

As part of a personalized medicine research program, The Genomedical Connection, a consortium between Duke University, the University of North Carolina at Greensboro, and the Moses Cone Health System in Greensboro, NC funded by the Department of Defense, developed a computerized self-administered FHH collection tool with integrated decision support, MeTree, to overcome barriers to the integration of FHH into primary care.

## Methods/Design

The Genomedical Connection developed a computerized self-administered FHH collection and decision support tool (MeTree) as part of a model for the practice of genomic medicine in primary care. This study was IRB approved by all three institutional partners as well as by the Department of Defense. The goal of the tool is to assist primary care providers in identifying high risk individuals who may need additional screening or referrals to maximize their preventative health care. It collects a 3 generation FHH on 48 conditions and currently provides decision support for 5 test conditions: breast cancer, ovarian cancer, colon cancer, thrombosis, and hereditary cancer syndromes. Diseases collected by the tool are shown in table 1. Decision support is provided in the form of printed reports. A patient report outlines important points for patients to discuss with their providers, while a provider report contains guideline recommendations for prevention and screening based upon estimated disease risk. Both receive a copy of the pedigree and the provider is given a tabular report of the family history as well. Examples reports are available in Additional File 1, Appendix A (provider report) and Additional File 2, Appendix B (patient report).

**Table 1:** Diseases collected by MeTree

Types of Cancer		
Brain	Lung	Small Bowel
Breast	Lymphoma	Stomach
Cervical	Melanoma	Testicular
Colon	Pancreatic	Thyroid
Kidney	Prostate	Uterine
Leukemia	Ovarian	Unknown
Liver	Skin (Not Melanoma)	Other, Specify

**Other Conditions**		

Alzheimer Disease/Dementia	Heart Attack	Multiple Sclerosis
Anemia	High Blood Pressure	Osteoporosis
Asthma	High Cholesterol	Parkinson Disease
Blood Clots in Veins	Inflammatory Bowel Disease	Rheumatoid Arthritis
Colon polyps	Lupus	Seizures
Diabetes	Macular Degeneration	Stroke
Glaucoma	Multiple Miscarriages	Thyroid Disease

**Hereditary Cancer Syndromes**		

Hereditary Breast & Ovarian Cancer (BRCA1/BRCA2 genes)		LiFraumeni Syndrome (TP53 gene)
Hereditary Nonpolyposis Colon Cancer (MLH1/MSH2/MSH6 genes)		Cowden Syndrome (PTEN gene)
Familial Adenomatous Polyposis (APC gene)		Other Cancer Syndromes

### Integration into clinic workflow

In order to minimize the burden on providers and the clinical staff, the MeTree tool is designed to have both a web and computer interface to permit access from the clinic for patients who do not have internet access at home. An introductory letter is provided to patients explaining the tool and the type of information they should collect from family members (along with a worksheet to complete) before attempting to use it; however, if they find that they cannot answer a question they are allowed to stop work and come back to it at a later date if necessary. Upon completion, the reports and pedigrees are printed by clinic staff with appropriate patient documents given to the patient and provider documents entered into the medical record to be available at the time of the appointment.

### Hybrid type 2 Effectiveness and Implementation Clinical Trial

Ultimately, the goal of MeTree is to help providers not only collect family history but to use it to detect those at high risk for disease who could benefit from alternative management strategies. To do this, the tool is designed to maximize sensitivity (especially around genetic counseling referrals for identification of hereditary cancer syndromes), understanding that it will be at the expense of a slightly lowered specificity. In order to assess the operating characteristics of the tool, to modify it to achieve the ideal combination of sensitivity and specificity, to measure its impact on patient and provider behaviors, and to identify factors critical to successful implementation in primary care, a hybrid type 2 trial of effectiveness and implementation is being conducted in 3 primary care clinics in the Moses Cone Health System. This type of hybrid study combines primary effectiveness outcomes with implementation outcomes, which are described in more detail in the outcomes section [5]. Two clinics serve as intervention sites and a third as a concurrent control to account for temporal trends in study outcomes.

Of the two intervention sites, one clinic has 8 board-certified internal medicine providers and 1 nurse practitioner while the other has 3 board-certified internal medicine physicians and 1 board-certified family medicine physician. Between the two sites there are 31,000 unique patient-visits annually and both have electronic medical records, onsite laboratories, and admitting privileges to Moses Cone Hospital. A computer with access to MeTree is available in a private room in each clinic. The control clinic has 6 board-certified family medicine physicians and 2 physician assistants. It serves ~20,000 patients annually and has an electronic medical record system.

### Recruitment

**Figure 1:**
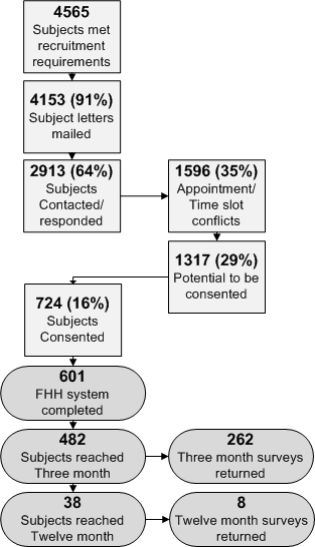
**Study flow diagram for patient recruitment and inclusion**.

All adult patients (over age 18) scheduled for an upcoming well visit in the 2 primary care practices are mailed an invitation to participate in the trial; only adoptees and non-English speakers are excluded. Those who meet these criteria and agree to participate (see Figure 1) are instructed by the study coordinators on how to use the tool and how to collect medical history from family members. They are also mailed the introductory letter and FHH collection worksheet (described in the *integration into clinical workflow *section). Participants arrive one hour early on the day of their provider appointment, are consented, and complete MeTree. Study coordinators are available to answer questions during the entire process, and upon completion, to print the appropriate documents for the patient and provider. Provider documents are scanned into the EMR by the nurse and the originals are handed to the provider as they enter the patient's room. It is left up to the providers and patients to act, or not, upon the recommendations as they deem appropriate.

In 9 months, 601 of the planned 1500 participants have been enrolled. Baseline characteristics of participants are similar to the general clinic population (table 2). Other measured characteristics include average family size of 10 +/- 5 (range 1-30) and an education level of: high school or less (14%), high school degree (63%), at least some graduate level courses (23%).

**Table 2:** Baseline characteristics of participants enrolled to date as compared to the general clinic population

	Study Participants	Baseline Clinic Population
Gender		

Male	192 (41.2%)	56.1%

Female	276 (58.8%)	42.7%

Ethnicity		

White	386 (82.4%)	61.7%

Black	66 (14.1%)	12.5%

Hispanic	10 (2.1%)	1.6%

Asian	1 (0.2%)	0.4%

Am. Indian/Eskimo	2 (0.4%)	0.4%

Ashkenazi Jewish	1 (0.2%)	0

Unknown	0	18.5%

Other	3 (0.6%)	1.6%

Age mean (sd)	58.2 (12.6)	59.3 (13.5)

### Outcomes and measures

The primary effectiveness outcomes are the change in 1) appropriate testing for hereditary thrombophilia, 2) appropriate referrals to genetic counseling for risk of hereditary cancer syndromes, and 3) appropriate screening rates for breast cancer, colon cancer, and ovarian cancer one year after using MeTree. The appropriateness of the risk management strategy recommended by MeTree and the risk management strategy recommended by the provider are determined by a genetic counselor after reviewing the pedigree. Secondary effectiveness outcomes, implementation outcomes, and covariates measured are shown in Table 3. Implementation is assessed using a mixed methods formative evaluation in order to identify barriers and potential solutions to those barriers that may potentiate broader adoption of the FHH system among primary care clinical settings. Examples of how formative evaluation results will be used include development of FAQs, modification of the tool interface and/or content, and development of resources to facilitate workflow changes.

**Table 3:** Study outcomes and measures

Variable	Measure	Source
**Primary Effectiveness Outcomes**		

Appropriate breast cancer screening	Risk-based guideline screening performed	Genetic counselor pedigree and chart review

Appropriate ovarian cancer screening	Risk-based guideline screening performed	Genetic counselor pedigree and chart review

Appropriate colon cancer screening	Risk-based guideline screening performed	Genetic counselor pedigree and chart review

Appropriate hereditary thrombophilia testing	Risk-based guideline screening performed	Genetic counselor pedigree and chart review

**Secondary Effectiveness Outcomes**		

Patient health-related behaviors	Diet Exercise Smoking status Intention to obtain cancer screening Knowledge about genetic testing	HINTS[16] given at baseline, 3 month and 12 months

Provider care patterns	Orders for: Screening studies Referrals to GC Genetic testing Documentation of: Disease risk MeTree recommendations	Chart review

MeTree operating characteristics	Sensitivity and specificity Net Reclassification Number needed to treat to identify high risk Subgroups affected by reclassification	Pedigree review by GC Pedigree review by PCP

Costs	Referrals, office visits, procedures, testing	Chart review

**Implementation Outcomes**		

Patient experience	Satisfaction Preparedness Ease of use Level of anxiety Time to use Questions\resource needs	Post-MeTree survey MeTree Study Coordinator

Provider experience	Agreement with recommendations Does it change practice Recommend it to peers Quality and usefulness of reports Impact on patient flow Time spent discussing MeTree reports	Provider survey and interviews

Clinic needs	Resource needs to implement Assistance required for patients/providers Training/time required for clinic staff	Study coordinators Interviews

**Covariates**		

Demographics	Age Gender Ethnicity	MeTree input

Education level		Baseline survey

Insurance		Baseline survey

Family	Size % with cancer	MeTree input

BMI		Measured at triage

Clinic		

Provider	Year of medical school graduation Gender	Provider survey

GC = genetic counselor; PCP = primary care provider

In the control clinic, rates of breast, colon, and ovarian cancer screening, thrombophilia testing, and genetic counselor referrals are assessed via chart review of 50 patients for each of the 7 providers, during two time periods: the year prior to the study start date and the year after study start date. Consecutive patients seen during the time period under evaluation are considered if they meet study entry criteria. Changes in the rates of screening between the two periods will serve as a guide for local temporal trends in screening rates and genetic counseling referrals and will be used to assist in the interpretation of changes seen in the intervention clinics.

### Analyses

The primary goals of this project are to measure the impact in the primary care setting of a structured FHH assessment tool on evidence-based prevention and screening for breast cancer, ovarian cancer, colon cancer, and thrombosis, and identification of hereditary cancer syndromes, as well as to measure the impact of implementation on patients, providers, and clinic processes.

For the effectiveness outcomes, we will calculate the proportion of subjects at the implementation clinics appropriately referred for genetic services (either to a genetic counselor or for a genetic test), or screening studies for breast, colon, and/or ovarian cancer. Although the study is implemented at the level of the clinical practice, the likelihood of clustering is low given that all participants undergo the intervention, the two clinical sites are similar in nature and serve similar patient populations within the same community, and the intervention is aimed at both the patient and the provider. To fully address the possibility that clustering may occur, we will calculate its design effect [6]; if the design effect is 1, we will use standard tests and generalized linear mixed models with clinic and provider as random effects, if not we will adjust the confidence intervals using a conditional logistic regression [7]. Effect size bias is extremely unlikely in this non-randomized study as all individuals receive the intervention, preventing the imbalance in treatment assignment that can lead to inaccurate point estimates [7].

### Sample size and Power

The empirical power for the comparison of referral rates between the implementation clinics and the control clinic will be a function of the rate in the control clinic at one year. For the purposes of power calculations we estimated the baseline referral proportion of 1% for genetic counseling, 77% for breast cancer screening, 59% for colon cancer screening based on a random sample of patient charts prior to launch of the MeTree tool in the implementation clinics and the CDC's Behavioral Risk Factor Surveillance System Survey data for North Carolina [8]. Based on this baseline percentage, using an alpha value of 0.025 to adjust for the comparisons with two clinics, a sample of 500 individuals will result in power of 80% for a screening proportion in the smaller implementation clinic of 0.147 or higher and a sample of 1000 individuals will result in power of 80% or higher for a screening proportion of 0.132 in the larger clinic. Alternatively if we don't consider the baseline proportion fixed but estimate it from the control clinic, we will be able to detect a difference in the proportions of 0.09 for the smaller clinic and 0.06 for the larger clinic with 80% power at an alpha of 0.025. Thus even analyzing each of the clinics separately we will have sufficient power to test the hypothesis that the MeTree tool implementation increased genetic referral rates.

## Discussion

The simplest and least expensive form of genetic assessment, family history, is widely overlooked in medical practice. It has long been taught as one of the core foundations of the medical interview, yet over the years these skills have been lost or overwhelmed by the pressures and time constraints of day to day practice. Bringing family history back into the realm of medical decision making is important for multiple reasons. First, disease prevention has the potential to reduce the ever escalating costs of medical care. However, disease prevention relies entirely upon accurate disease risk prediction. Family history has been shown to improve disease risk prediction for colon cancer [9], breast cancer [10], and cardiovascular disease [11], among others. Second, using family history to predict disease requires several skills: understanding what family history is saying about risk, understanding how to communicate risk to patients, and using risk information to motivate behavior change. By developing an electronic family history collection and decision support tool to assist providers, we anticipate that providers will be able to reincorporate family history based decision making into their medical practice. In addition, continued use may permit providers to establish and hone their skills in a way that they can start to apply them to other risk-based fields, thus it may serve as an educational stepping stone to newer and more complicated areas of genetics and genomics.

Despite these benefits of family history taking little is known about its clinical validity or utility [12]. Our study fills this significant gap by directly addressing the question of both validity and utility. Although the one year follow up will not permit the use of hard outcomes such as the incidence of breast, colon, or ovarian cancer, screening is indicated by the United States Preventive Task Force and the American Cancer Society because of evidence that screening reduces morbidity and mortality; but is considerably underutilized [13,14]. With this in mind screening patterns may be used as an acceptable intermediate endpoint.

Another important gap our study addresses is implementation. Multiple barriers to implementation exist. At the patient level inaccurate or limited information about family history is widespread [12]. At the provider level, as mentioned above, time constraints and lack of training are becoming more and more difficult to address [15]. Developing an electronic tool for collecting family history and providing decision support at the point of care are essential keys to addressing these barriers and our tool, MeTree does just that; however integrating it into practice creates its own set of barriers. Technology can be a barrier for both patients and providers, as can the resources required to integrate it. We monitor all stakeholders and measure every aspect of the implementation process in order to get a clear picture of what barriers have been solved with MeTree and what new ones arise in their place. At the end of this study we expect to have an excellent idea of not only whether such a tool has value in clinical practice but also how best to facilitate widespread adoption. Ultimately, it may serve as a demonstration project to guide integration of other personalized medicine tools, such as health risk assessments, into clinical practice.

## Competing interests

The authors declare that they have no competing interests.

## Authors' contributions

All of the authors participated in the design and implementation of the study as well as contributed to the drafting of the manuscript. EH is responsible for the statistical analyses. All authors read and approved the final manuscript.

## Pre-publication history

The pre-publication history for this paper can be accessed here:

http://www.biomedcentral.com/1472-6963/11/264/prepub

## Supplementary Material

Additional file 1 **Example of a Provider Report**. Shows the type of information a provider report contains and its layout. Click here for file

Additional file 2 **Example of a Patient Report**. Shows the type of information a patient report contains and its layout. Click here for file
