# Supplementary figures and images for: Protocol for implementation of family health history collection and decision support into primary care using a computerized family health history system
Source: BMC Health Serv Res. 2011 Oct 11;11:264. doi: 10.1186/1472-6963-11-264 (PMC3200182; doi:10.1186/1472-6963-11-264)

**Appendix A**. Example provider report


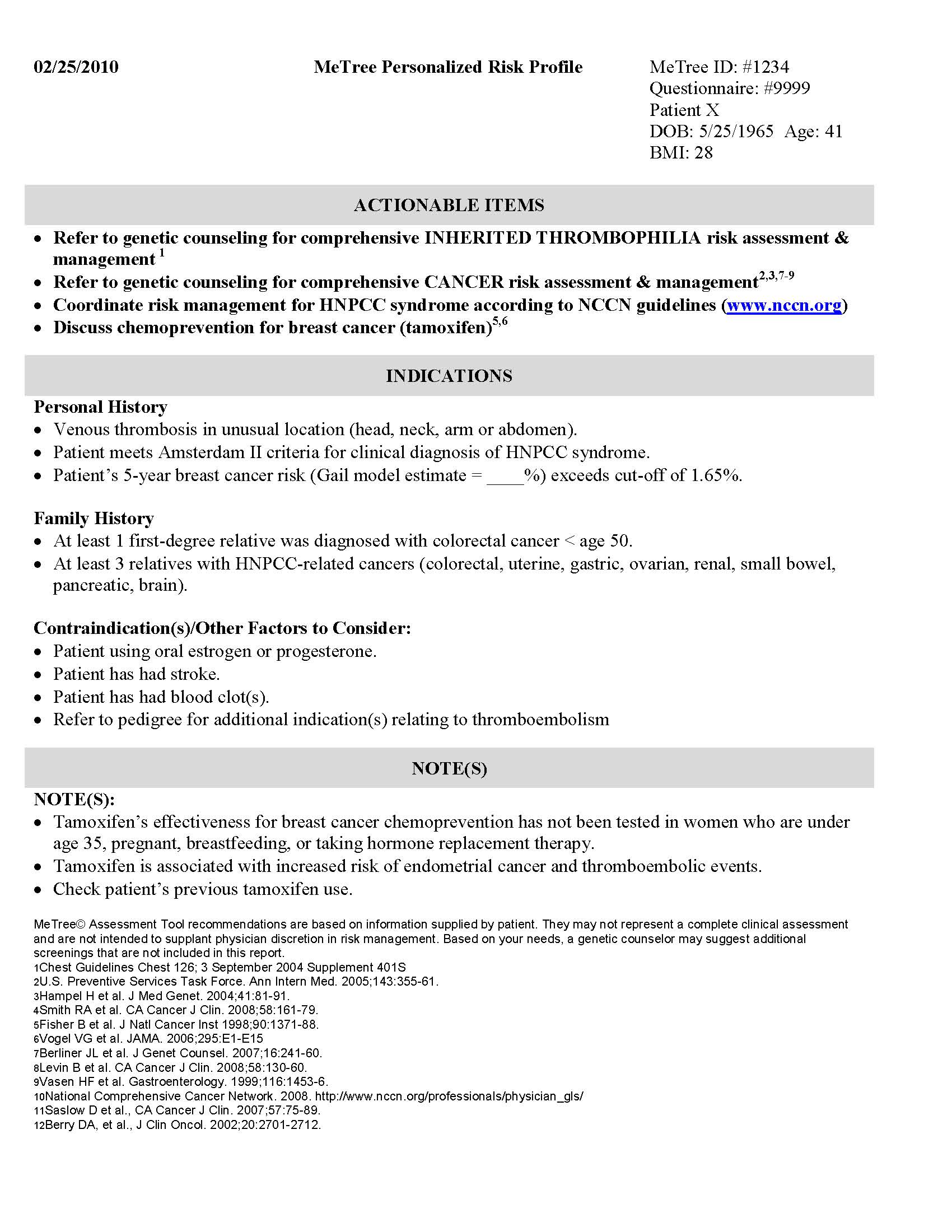

Supplement: Additional file 1 — Example of a Provider Report. Shows the type of information a provider report contains and its layout. [file 1472-6963-11-264-S1.DOCX]

**Appendix B**. Example Patient Report


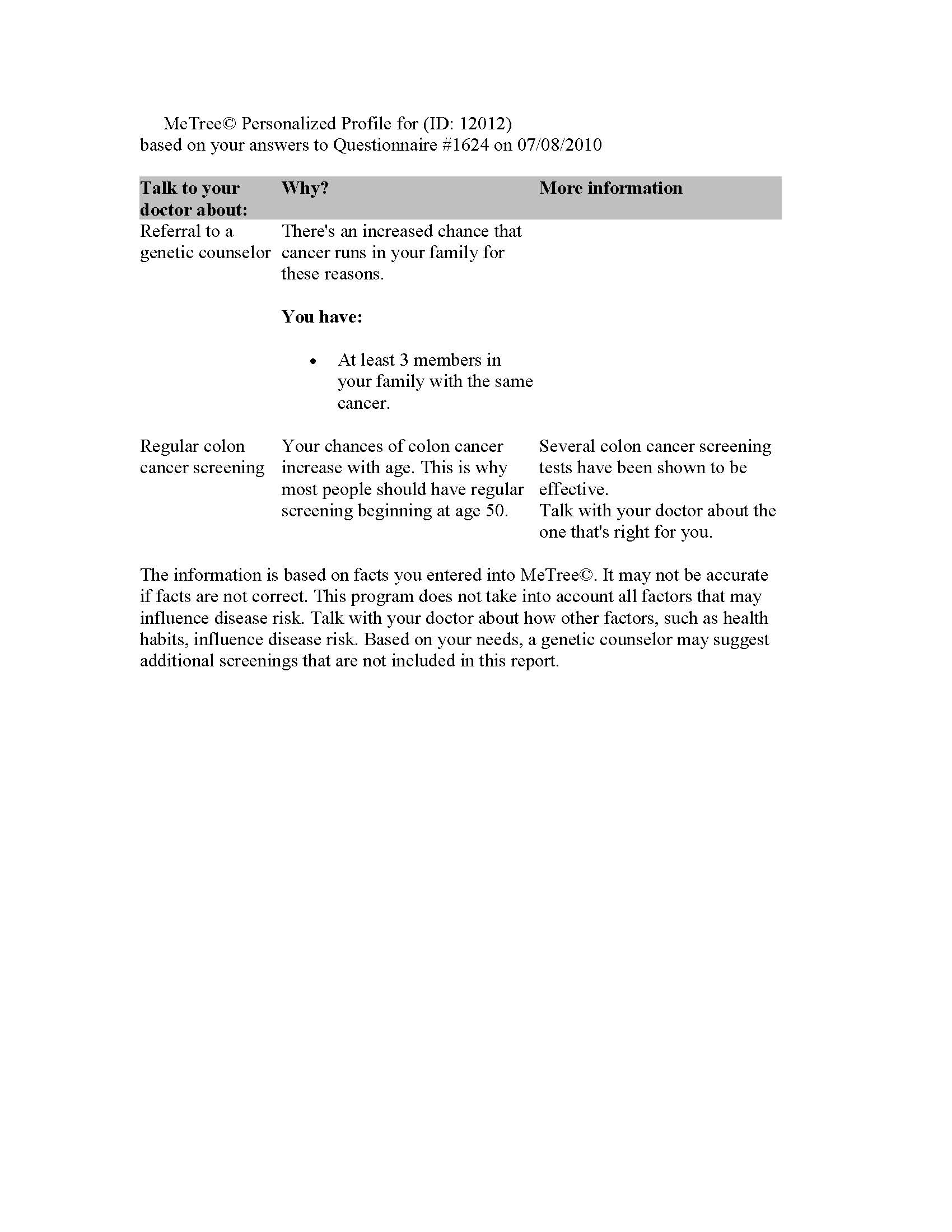

Supplement: Additional file 2 — Example of a Patient Report. Shows the type of information a patient report contains and its layout. [file 1472-6963-11-264-S2.DOCX]
